# Similar and Divergent Roles of Stringent Regulator (p)ppGpp and DksA on Pleiotropic Phenotype of Yersinia enterocolitica

**DOI:** 10.1128/spectrum.02055-22

**Published:** 2022-11-21

**Authors:** Can Huang, Jiao Meng, Wenqian Li, Jingyu Chen

**Affiliations:** a Beijing Laboratory for Food Quality and Safety, College of Food Science & Nutritional Engineering, China Agricultural University, Beijing, People’s Republic of China; b Laboratory of Nutrient Resources and Synthetic Biology, Tianjin Institute of Industrial Biotechnology, Chinese Academy of Science, Tianjin, People’s Republic of China; Penn State University

**Keywords:** *Yersinia enterocolitica*, (p)ppGpp, DksA, stringent response, stress resistance

## Abstract

Stringent response plays an important role in the response of *Enterobacteriaceae* pathogens to rapid environmental changes. It has been shown that synergistic and antagonistic actions exist between the signaling molecules (p)ppGpp and DksA in several foodborne pathogens; however, the biological function of these molecules and their interactions in *Yersinia* are still unclear. This study systematically investigated the role of stringent response in Yersinia enterocolitica, a typical foodborne *Enterobacteriaceae* pathogen, by deleting the (p)ppGpp and DksA biosynthesis genes. (p)ppGpp and DksA copositively regulated most phenotypes, such as motility, antibiotic resistance, and tolerance to oxidative stress, whereas they exhibited independent and/or divergent roles in the growth and biofilm synthesis of Y. enterocolitica. Gene expression analysis revealed that (p)ppGpp- and DksA-deficiency reduced the transcription of flagellar synthesis genes (*fliC* and *flgD*) and biofilm synthesis genes (*bssS* and *hmsHFRS*), which could potentially contribute to changes in motility and biofilm formation. These results indicate that stringent response regulators (p)ppGpp and DksA have a synergistic role and independent or even completely opposite biological functions in regulating genes and phenotypes of Y. enterocolitica. Our findings revealed the biofunctional relationships between (p)ppGpp and DksA and the underlying molecular mechanisms in the regulation of the pathogenic phenotype of Y. enterocolitica.

**IMPORTANCE** The synergetic actions between the stringent response signaling molecules, (p)ppGpp and DksA, have been widely reported. However, recent transcriptomic and phenotypic studies have suggested that independent or even opposite actions exist between them. In this study, we demonstrated that the knockout of (p)ppGpp and DksA affects the polymorphic phenotype of Yersinia enterocolitica. Although most of the tested phenotypes, such as motility, antibiotic resistance, and tolerance to oxidative stress, were copositively regulated by (p)ppGpp and DksA, it also showed inconsistencies in biofilm formation ability as well as some independent phenotypes. This study deepens our understanding of the strategies of foodborne pathogens to survive in complex environments, so as to provide theoretical basis for the control and treatment of these microorganisms.

## INTRODUCTION

When the external environment changes, bacteria rapidly reintegrate intracellular resources and energy by stopping the synthesis of DNA, stable RNA and membrane components and rapidly synthesizing factors that are important for stress resistance, thereby ensuring their survival (1). This so-called stringent response is cooperatively controlled by two intracellular signaling molecules, guanosine pentaphosphate and guanosine tetraphosphate (collectively referred to as [p]ppGpp), and DksA. As a global regulatory system, stringent responses are involved in regulating bacterial virulence, motility, and tolerance to multiple antibiotics (1, 2). During exponential growth, the stringent response is also present at basal levels and functions to modulate bacterial growth rates and adjust metabolic levels (3, 4).

There are three common nucleotide-based secondary messengers in bacteria: cyclic AMP, cyclic di-GMP, and (p)ppGpp (2). The concentration of these secondary messengers changes in response to external environmental stimuli, adapting bacteria to stressful conditions by modulating transcriptional levels at target sites. Transcriptome analysis showed that the stringent response could regulate 16 to 26% of all genes involved in synthesizing DNA, tRNA, ribosomal proteins, fatty acids, amino acids, and membrane components (5–7). Cellular (p)ppGpp levels are mainly controlled by RelA-SpoT homolog (RSH) enzymes, which are highly conserved in bacteria and are not found in a few species, including *Verrucomicrobia, Chlamydiae*, and *Planctomycetes* (8). In Gram-negative enteric bacteria, two long-RSH enzymes, RelA and SpoT, control the cellular pool of (p)ppGpp. Both have synthetic activity using ATP and either GTP or GDP to generate (p)ppGpp; however, only SpoT can hydrolyze (p)ppGpp to yield pyrophosphate and either GDP or GTP because of the absence of hydrolytic activity in the RelA hydrolysis domain (HD). In contrast, Gram-positive bacteria express a single short-RSH protein, Rel, with both synthetic and hydrolytic activities. Unlike SpoT, Rel shows similar high synthetic activity to RelA in response to amino acid starvation (1, 2). Furthermore, single-domain, monofunctional RSHs, including small alarmone synthetases (SASs) and small alarmone hydrolases (SAHs) have been discovered in various bacterial species, such as Bacillus subtilis (9) and Mycobacterium smegmatis (10).

(p)ppGpp exerts transcriptional and physiological effects through indirect or direct mechanisms. Direct mechanisms of action are achieved by binding to target proteins. This includes the regulation of enzyme activity through interaction with RNA polymerase (RNAP) in cooperation with DksA. The positive or negative effects of (p)ppGpp and DksA are determined by the properties of involved promoter (11). In addition, (p)ppGpp can inhibit protein synthesis by inhibiting ribosomal translation factors (such as IF2, EF-Tu, and EF-G) and phosphate metabolism by binding to polyphosphate kinase (PPK) (12–15). Indirect mechanisms require the involvement of other regulatory factors. An important target is the transcriptional repressor CodY, which can be activated by binding GTP and regulating more than 100 target genes involved in adaptation to stress, thereby affecting bacterial phenotypes such as proliferation ability, flagella synthesis, sporulation, and virulence (16). (p)ppGpp can also regulate transcription by altering sigma factors that bind to RNAP. During a stringent response, high (p)ppGpp concentrations inhibit RNAP binding to strong σ^70^-dependent promoters and release more RNAP to bind to the alternative σ-factors, then direct RNAP to regulate target gene transcription (1). Furthermore, it has been reported that a dialogue between (p)ppGpp and cyclic di-GMP (c-di-GMP) enables bacteria generate persister cells and cope with environmental pressures (17).

In addition to (p)ppGpp, the 17-kDa transcription factor DksA play a crucial role in the stringent response. Based on bioinformatic alignment, it is generally believed that DksA is present in most proteobacteria species but not in *Firmicutes* or *Thermophilic*. However, it should be emphasized that this statement is not definitive due to the incomplete understanding of the function and structure of DksA homologs (18). It was reported that DksA regulated about 7% and 20% of all genes in Escherichia coli and Xanthomonas citri, respectively (5, 6). Both direct and indirect regulatory mechanisms of DksA also exist. The best-known indirect regulatory mechanism is sigma factor competition, as described above. In addition, DksA can regulate promoter of small RNAs regulated by ppGpp/DksA and indirectly influence target genes. It has also been reported that DksA can directly activate *ssrB* gene expression, thereby facilitating balanced SPI2 (Salmonella pathogenicity island-2) virulence gene transcription (19). In Xanthomonas citri, DksA is involved in histidine metabolism, TonB-dependent transporters, type 2 secretion system (T2SS), and type 3 secretion system (T3SS), indicating the importance of DksA in nutrition uptake, host adaptation and virulence (6).

DksA acts as a cofactor to enhance ppGpp transcriptional regulation. Several studies have reported synergistic actions between them, including an anti-oxidation effect (20). However, recent studies have suggested that antagonistic actions also exist between DksA and (p)ppGpp (21, 22). The primary evidence is that DksA-and ppGpp-deficient strains show opposite effects phenotypically and transcriptionally, e.g., flagellar assembly gene (6). Moreover, DksA overproduction can also compensate for specific traits in the absence of (p)ppGpp (23). These complex and diverse regulatory patterns are responsible for the stability of the intracellular environment in variable external environments. Thus, unraveling the interaction provides a deeper understanding of the stringent response mechanism and has important implications for revealing bacterial lifestyles.

Stringent response not only helps bacteria to quickly adapt to the stressful environment, but also affects the virulence. For foodborne pathogens, several reports have shown regulation of stringent response signaling molecules (p)ppGpp and DksA on bacterial virulence-related phenotypes, such as S. enterica serovar Typhi (24), E. coli (25), S. aureus (26). The impact of (p)ppGpp and DksA has yet been studied in Y. enterocolitica. In this study, we examined the phenotypes associated with bacterial pathogenicity in Y. enterocolitica DksA- and (p)ppGpp-deficient strains and focused on the differences in the regulatory mechanisms of the two signaling molecules. Using phenotypic characterization and target gene expression determination, this study provides new insights into the function of stringent response regulators and interactions between (p)ppGpp and DksA in Y. enterocolitica.

## RESULTS

### (p)ppGpp is required for Y. enterocolitica growth in an oligotrophic environment.

In general, nutrient-adequate conditions required for bacterial growth are only available under laboratory conditions, and in the natural environment bacteria need to quickly adapt to nutrient-poor conditions to ensure their survival and spread. To explore in more detail the effect of Y. enterocolitica stringent response regulators (p)ppGpp in a low nutrient environment, we constructed the Δ*relA* strain (designated YENR) and Δ*relA*Δ*spoT* strain (designated YENRS) based on wild-type strain Y. enterocolitica ATCC 23715 (Table 1). Although several attempts have been made, the Δ*spoT* strain was not obtained, which might be caused by excessive accumulation of intracellular (p)ppGpp (27, 28). Moreover, complementary strains were obtained by transforming the pBAD24-based plasmid, and the inducible PBAD promoter controlled gene expression. All assays in this study were conducted with the addition of 0.02 g/L l-arabinose if without any other declares to make data comparable. Real-time quantitative PCR (RT-qPCR) experiments showed that the expression levels of *relA* and *spoT* in YENRS and *relA* in YENR were undetectable but returned to a relatively stable level through plasmid complementation (Fig. S1A).

**TABLE 1:** Strains and plasmids used in this study^*a*^

Strains and plasmids	Relevant characteristics	Sources
E. coli		
DH5α	F-, φ80*lacZ*ΔM15, Δ(*lacZYA*-*argF*)*U169*, *deoR*, *recA1*, *endA1*, *hsdR17*(rk-, mk+), *phoA*, *supE44*, λ-, *thi-1*, *gyrA96*, *relA1*	Lab stocked
S17-1λpir	*recA1, thi, pro, hsdR-*M*^+^,* RP4:2-Tc:Mu-Kan:Tn7, λpir	Lab stocked
Y. enterocolitica		
ATCC 23715	WT, serotype O:8, Biotype 1B, pYV-	Lab stocked
YEND	Δ*dksA*	This study
YENR	Δ*relA*	This study
YENRS	Δ*relA*, Δ*spoT*	This study
YENDRS	Δ*dksA*, Δ*relA*, Δ*spoT*	This study
YEND-D	Δ*dksA*, P*_BAD_dksA*; Amp^r^	This study
YENR-R	Δ*relA*, P*_BAD_relA*; Amp^r^	This study
YENRS-S	Δ*relA*, Δ*spoT*, P*_BAD_spoT*; Amp^r^	This study
Plasmids		
pDS132	Conditional replication vector; R6K origin, mobRK4 transfer origin, sucrose-inducible-*sacB*; Cm^r^	Lab stocked
pBAD24	*AraC*, promoter P*_BAD_*; Amp^r^	Lab stocked
pDS132-Δ*dksA*	Upstream and downstream *dksA* fragments were cloned into pDS132; Cm^r^	This study
pDS132-Δ*relA*	Upstream and downstream *relA* fragments were cloned into pDS132; Cm^r^	This study
pDS132-Δ*spoT*	Upstream and downstream *spoT* fragments were cloned into pDS132; Cm^r^	This study
pBAD24-*dksA*	P*_BAD_ dksA*; Amp^r^	This study
pBAD24-*relA*	P*_BAD_ relA*; Amp^r^	This study
pBAD24-*spoT*	P*_BAD_ spoT*; Amp^r^	This study

a Amp, ampicillin; Cm, chloramphenicol; r, resistance.

**FIG 1:**
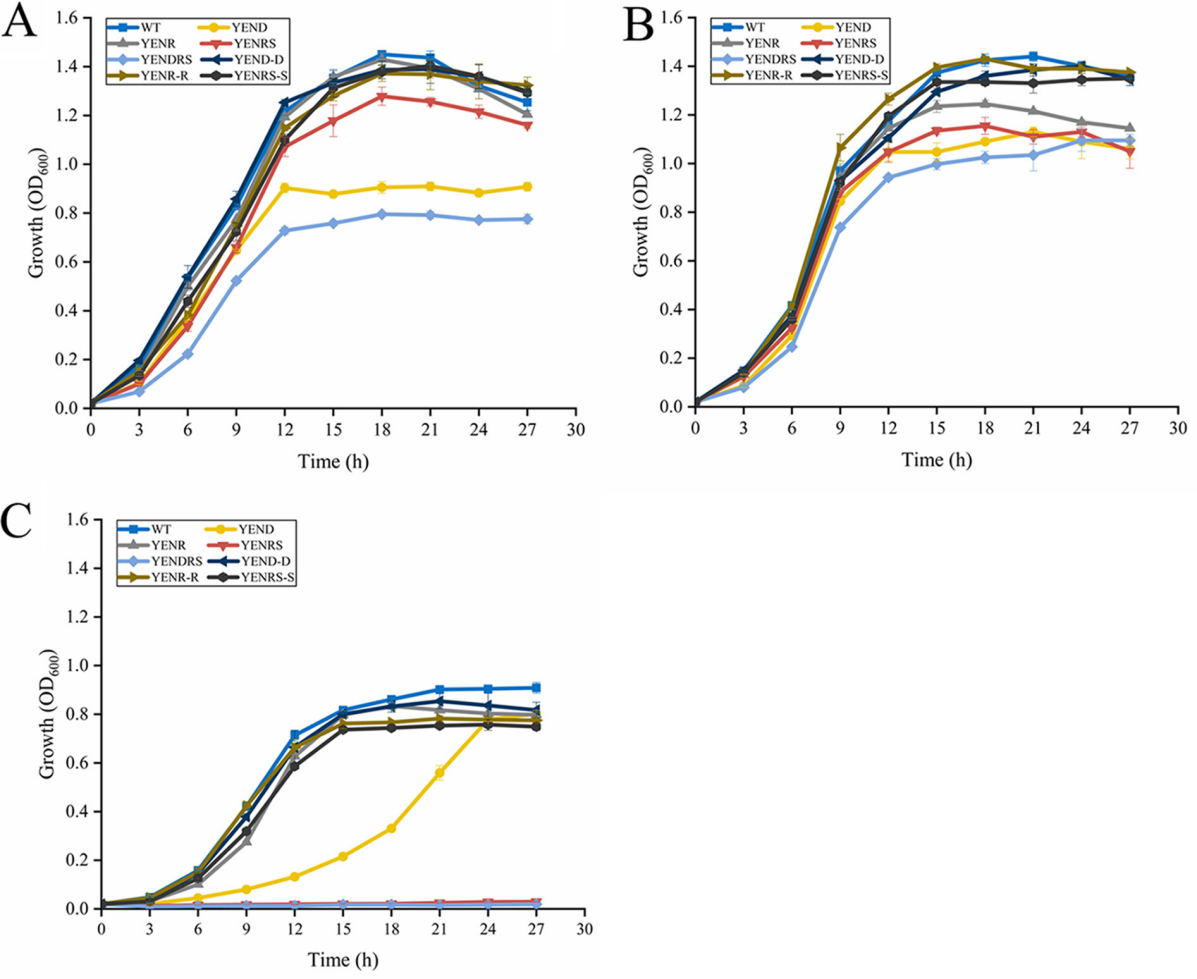
Growth characteristic of WT and mutant strains in LB (A), LBNS (B), and M63 minimum medium (C) supplemented with 0.02% l-arabinose. Data are mean OD_600_ for three independent cultures and standard errors of the means.

Growth rates of the wild type (WT), YENR, and YENRS strains in rich lysogeny broth (LB), LBNS (LB without salts), and M63 minimal media were determined (Fig. 1A to C). Compared to the WT, YENRS showed a longer lag phase and slightly weaker growth rate in LB and LBNS media, and even completely impaired growth in M63 minimal medium, indicating that the growth of Y. enterocolitica was dependent on (p)ppGpp synthesis in an oligotrophic environment. Interestingly, *relA* single deletion mutant showed roughly the same growth curve as the WT, rather than an absolute loss of growth ability as in YENRS. Similar results were observed in YENRS strains complemented by the *spoT* gene. Considering *spoT* possesses (p)ppGpp synthesis and hydrolysis activities, we speculated that SpoT could synthesize sufficient (p)ppGpp in Y. enterocolitica to cope with auxotrophic conditions.

### (p)ppGpp contributes to bacterial resistance to multiple environmental stresses.

**FIG 2:**
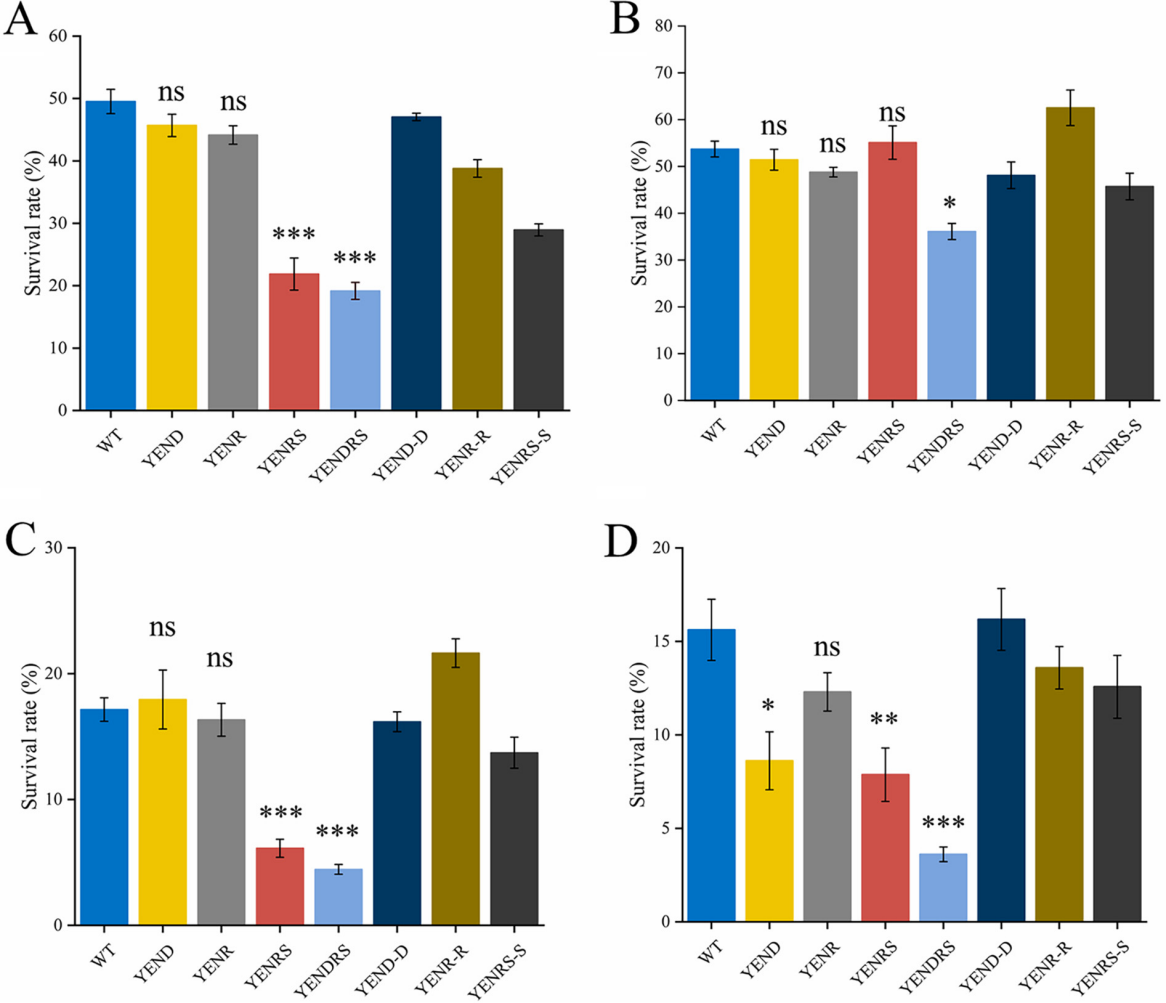
The role of DksA and (p)ppGpp in stress resistance. Survival rate of WT, mutant strains and the complemented strain after challenge with pH 4.0 (A), pH 10.0 (B), or 0.5 M NaCl (C) for 60 min, or 1 mM H_2_O_2_ for 30 min (D) were determined. Data are mean ± SD of three biological repeats, each of which was perforemed with three technical replicates. An asterisk indicates a significant difference with ns, not significant; *, *P* < 0.01; **, *P* < 0.001; ***, *P* < 0.0001.

Previous studies have suggested that stringent responses play a role in bacterial resistance to multiple environmental stresses (29). For the foodborne pathogen Y. enterocolitica, tolerance to acids, alkalis, high osmosis, and oxidants is very important for the strain to adapt to complex food environments quickly (29, 30). Therefore, we assessed the survival rate of the WT and mutant strains under these environmental stresses (Fig. 2 A to D). After 1 h of LBNS (pH 4.0) treatment, the survival rate of the YENRS mutant was 21.87%, whereas that of the WT strain was 49.52%, indicating that (p)ppGpp plays an essential role in coping with acid stress in Y. enterocolitica. Furthermore, complementation with the pBAD24-*spoT* plasmid restored the acid resistance to a certain extent. The survival rate of the YENR mutant was similar to that of the WT strain, which may be ascribed to the (p)ppGpp synthesis activity of SpoT. A similar phenomenon was observed in the WT and mutant strains after treatment with high osmotic pressure (LBNS supplemented with 0.5 M NaCl). The survival rate of the YENRS mutant was approximately one-third that of the WT strain, which can be complemented by the survival rate of YENR by expressing SpoT induced by l-arabinose. Treatment with oxidative stress conditions resulted in a half survival rate decrease in YENRS mutant (7.87%) compared to the WT strain (15.62%). Neither the YENR nor YENRS mutants showed an evident decrease in resistance to base stress (pH 10.0). These results confirmed that (p)ppGpp contributes to bacterial resistance to acid and high osmotic and oxidative stress in *Y. enterocolitis*.

### (p)ppGpp plays a role in *Y. enterocolitis* motility and biofilm formation.

**FIG 3:**
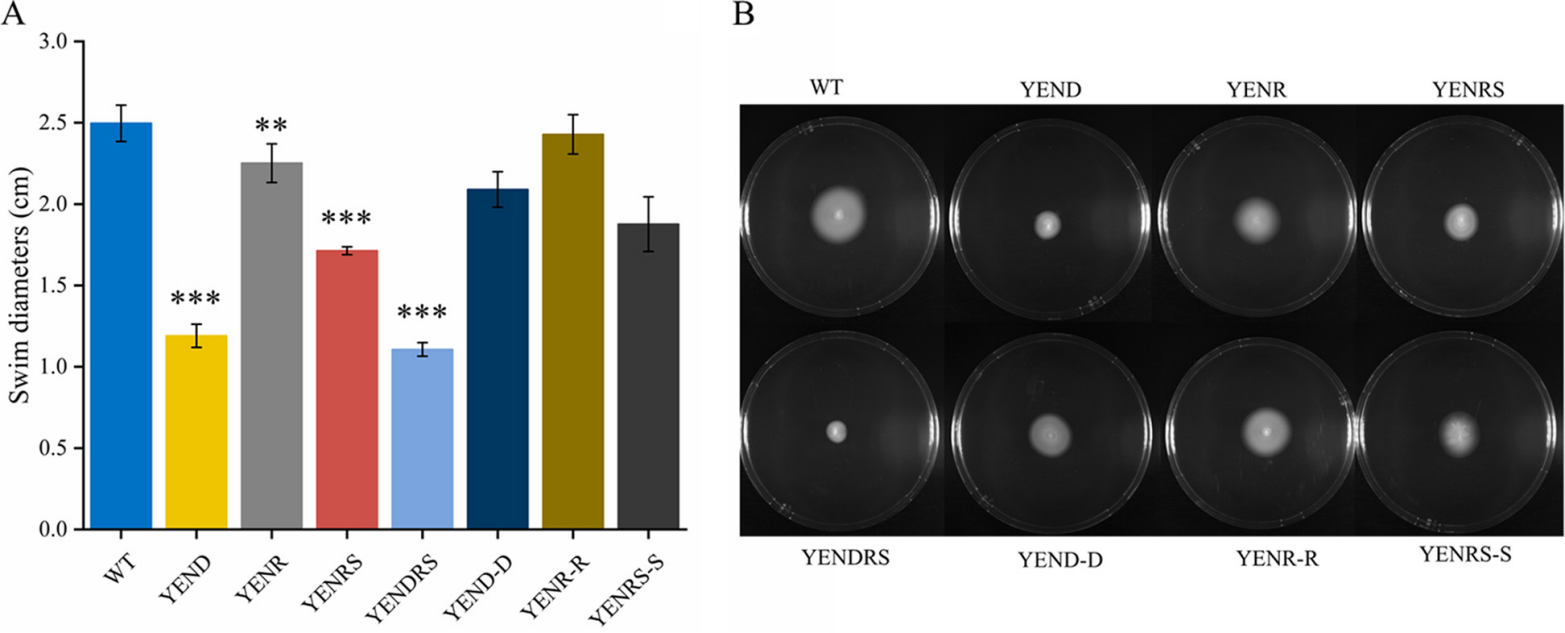
Motility assay of WT, mutant strains and the complemented strain in 0.35% LBNS agar plate. A 1.0-μL of each Y. enterocolitica *culture* was injected into the plate. The plates were incubated at 26°C for 48 h before photographing. (A) Quantification of swim diameters. (B) Images of swim plate. The results are mean of four independent plates, and the error bars indicate standard deviations. An asterisk indicates a significant difference with **, *P* < 0.001; ***, *P* < 0.0001.

**FIG 4:**
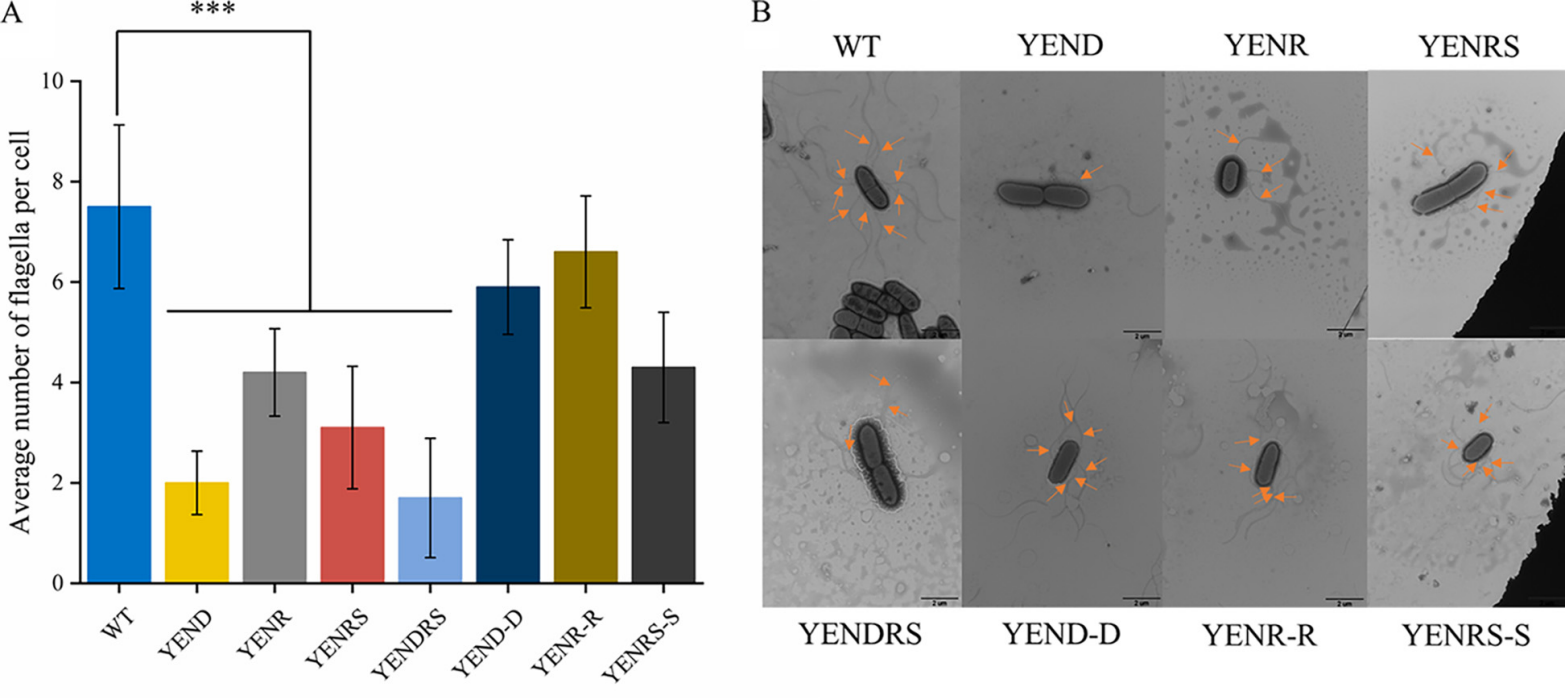
Flagella biosynthesisof WT, mutant strains and the complemented strain. Strains grown to the midlog phase in LBNS medium were stained with phosphotungstic acid and the flagella were visualized and the average flagella numbers in a single cell were calculated. (A) Average number of flagella per cell in various strains. The data are presented as the mean ± SD of at least three biological repeats, and the error bars indicate standard deviations. An asterisk indicates a significant difference ***, *P* < 0.0001. (B) Transmission electron microscopy pictures of the wild-type, mutant strains and the complemented strain. The scale bar represents 2 μm and the orangearrow in the picture refers to the flagella.

Motility and biofilm formation are two phenotypes closely related to the virulence of pathogens *in vivo*. Previous studies suggested that deletion of *relA* and *spoT* in Proteobacteria would lead to motility deficiency and changes in biofilm formation (31, 32); therefore, the swim diameter of *Y. enterocolitis* WT and mutants in LBNS plates and their biofilm formation ability in 24-well polycolor microtiter plates were analyzed. As shown in Fig. 3, the swim diameter of YENRS mutants decreased by approximately 31% and could be partially complemented by introducing the pBAD24-*spoT* plasmid. In addition, a mild but significant reduction of 10% was observed in YENR compared to in the WT strain. Complementation with the *relA* gene restored the impaired motility of the YENR strain to the level of the WT background. Flagella is an important motor organ of bacteria, and its quantity is positively correlated with bacterial motility ability, which plays a key role in the pathogenicity and competitiveness of bacteria. The change in its quantity is positively correlated with bacterial motility, which is also presumed to contribute to the pathogenicity and competitiveness of pathogens. Therefore, flagella formation in the WT, mutant, and complement strains was examined using electron microscopy (Fig. 4). The results showed that the WT strain had about 7.5 flagella per cell, and the YENRS mutant had weakened flagella synthesis with an average of 3.1 flagella per cell. Moreover, expressing *spoT* in the YENRS mutant can partly restore the flagellar number. The results showed that the loss of (p)ppGpp resulted in a deficiency in motility and reduction in flagellum synthesis in *Y. enterocolitis*.

**FIG 5:**
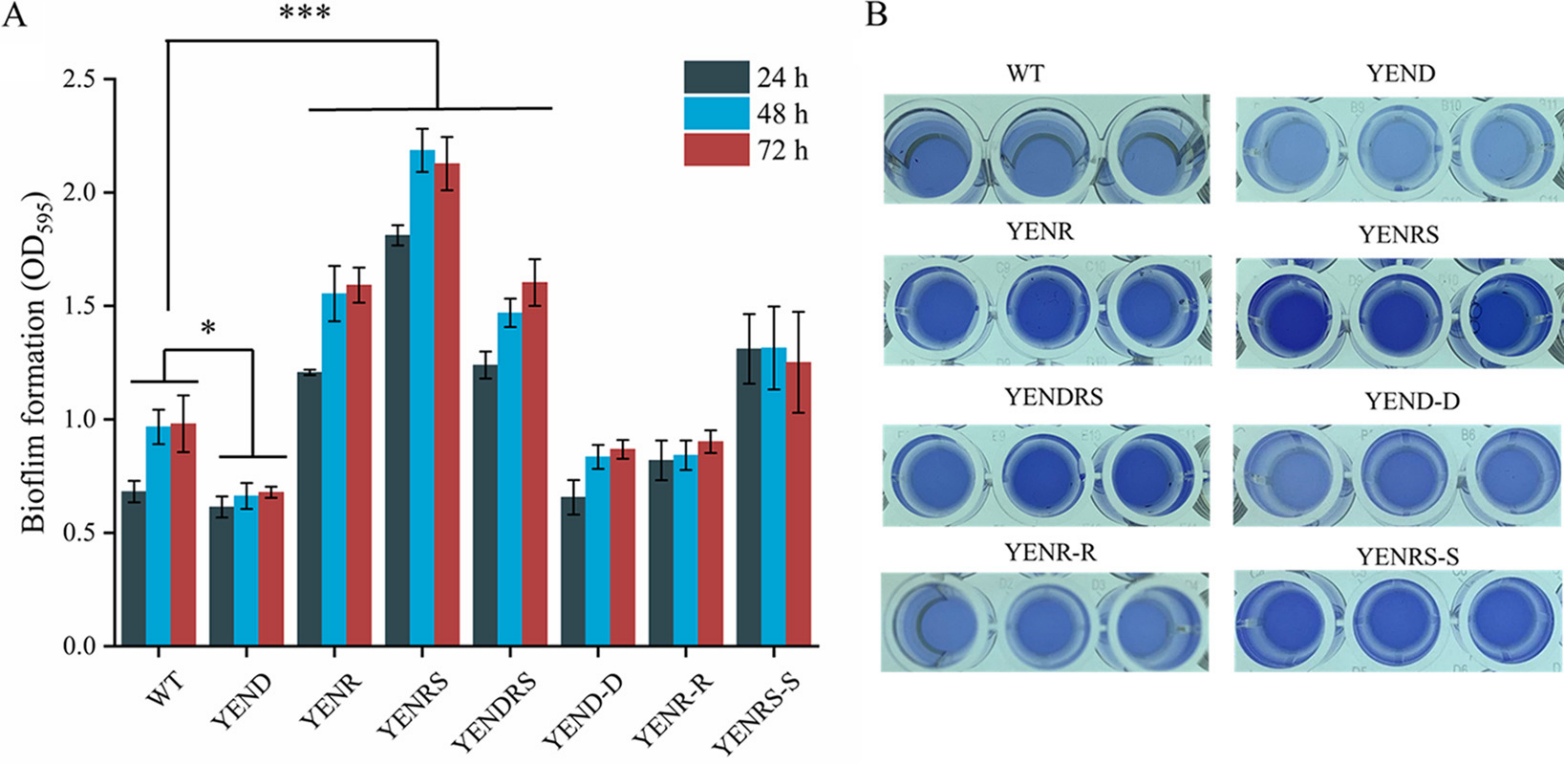
Biofilm formation of WT, mutant strains and the complemented strain in LBNS medium. Y. enterocolitica were cultured in LBNS media at a 24-well polystyrene microtiter plate. The biofilms were stained with crystal violet after 24, 48 and 72 h of incubation and measured at 595 nm. (A) Quantification of biofilm. (B) Images of purple color depth after 72 h of incubation. Data are mean of six biological repeats, and the error bars indicate standard deviations. An asterisk indicates a significant difference with *, *P* < 0.01; ***, *P* < 0.0001.

**FIG 6:**
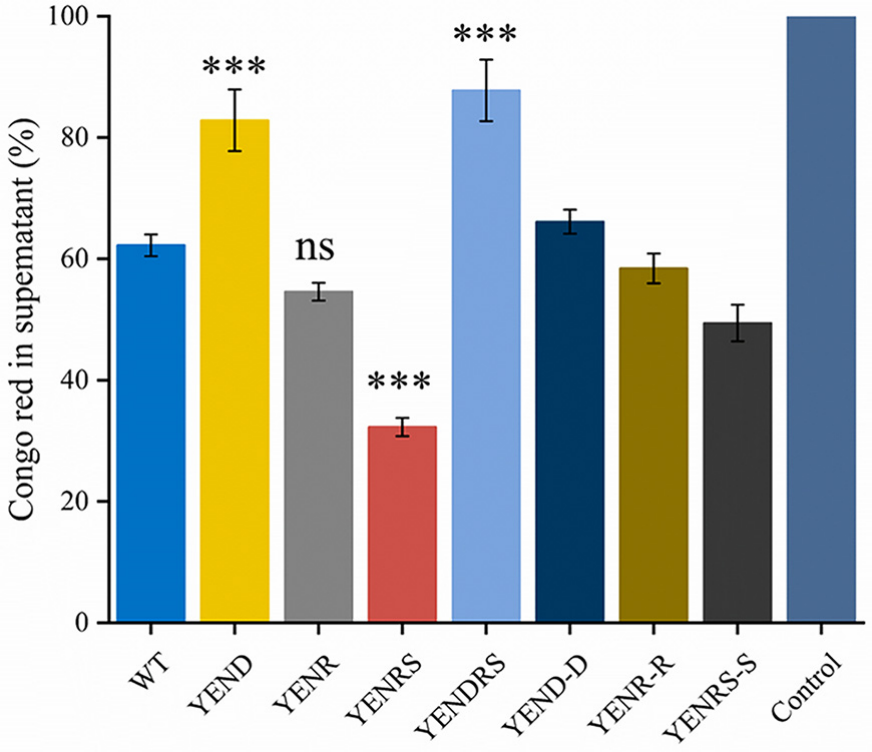
Congo red binding assay of WT, mutant strains and the complemented strain. The mass of Y. enterocolitica was mixed with Congo red for binding. After removing bacterial mass, the optical density of Congo red in supernatant was measured at 490 nm, each of which was normalized by relating to the value of 100 μg/mL Congo red at 490 nm. Data are mean of three biological repeats, and the error bars indicate standard deviations. An asterisk indicates a significant difference with ns, not significant; *, *P* < 0.01; **, *P* < 0.001; ***, *P* < 0.0001.

The formation and development of bacterial biofilms are important causes of damage to food production equipment, product contamination, and medical infections. To examine the role of (p)ppGpp in biofilm formation, we quantified the biofilm amounts of the WT, YENR, YENRS, YENR (pBAD24-*relA*), and YENRS (pBAD2- *poT*) strains by crystal violet staining at 24, 48, and 72 h in 24-well polystyrene microtiter plates (Fig. 5). Interestingly, knocking out (p)ppGpp nearly doubled Y. enterocolitica biofilm production, whereas the complement of *spoT* in the YENRS strain significantly reduced biofilm formation to a level close to that of YENR. In addition, the deletion of *relA* increased the biofilm formation slightly from 0.98 to 1.25 at 72 h. Biofilm is defined as a structured community mainly composed of exopolysaccharides (EPSs), cells, and fibrin attached to a contact surface (33). Food, food processing equipment, and pipelines are the best sites for microbial attachment and biofilm formation. Therefore, we also assessed the relative amount of EPS in the WT, mutant, and complementary strains using Congo red assays. As shown in Fig. 6, Congo red left of YENRS in the supernatant was lower than that of the WT strain, indicating that more EPS was synthesized and secreted by the (p)ppGpp-deficient strain. All these results of the Congo red assays maintain the same tendency of biofilm formation. From these experiments, we concluded that (p)ppGpp can inhibit biofilm formation and EPS synthesis in *Y. enterocolitis*.

### (p)ppGpp confers higher chloramphenicol and ampicillin resistance to Y. enterocolitica.

(p)ppGpp is a potential target for developing broad-spectrum bacteriostatic agents owing to its distinct absence in humans and intricate regulation of catalytic activity (34). To investigate the effect of (p)ppGpp on the resistance of Y. enterocolitica to different classes of antibiotics, including polypeptides, amido alcohol, and penicillin, the MIC of polymyxin B, chloramphenicol, and ampicillin were determined using the broth 2-fold dilution method. As shown in Table 2, there was no distinction among the WT, mutant, and complement strains when treated with polymyxin B at an MIC of 1 μg/mL. However, chloramphenicol exhibited MIC values equivalent to 4 and 2 μg/mL against the WT and YENRS strains, respectively. Notably, the MIC of chloramphenicol against YENR was also 2 μg/mL, indicating that RelA, instead of SpoT, is a potential target for chloramphenicol resistance. Only the WT and mutant strains were tested for ampicillin resistance gene expression in pBAD24. The results indicated that the MIC values against the YENRS strain were a quarter of those against the WT strain, and the deletion of RelA reduced the MIC values by half. In summary, (p)ppGpp confers Y. enterocolitica with higher chloramphenicol and ampicillin resistance.

**TABLE 2:** Antibiotic susceptibility of WT, mutant strains and the complemented strain

	MIC (μg/mL) Chloramphenicol	MIC (μg/mL) Ampicillin	MIC (μg/mL) Polymyxin B
WT	4	16	1
YEND	0.5	4	1
YENR	1	8	1
YENRS	1	4	1
YENDRS	0.5	4	1
YEND-D	2	ND	1
YENR-R	2	ND	1
YENRS-S	1	ND	1

### DksA and (p)ppGpp showed opposite effects on growth and biofilm production in Y. enterocolitica.

DksA binds to and widens the RNAP secondary channel as a stringent response factor, thereby influencing core orientation and shelf modules (2). Moreover, a few studies have suggested that the RNAP-DksA interaction probably amplifies the signal from (p)ppGpp during transcription elongation, thereby synergistically regulating bacterial phenotypes, such as growth and response to external environmental stress (35). However, some studies have found that the regulatory mechanisms of DksA and (p)ppGpp are separate, possibly because of their opposite binding surfaces on RNAP (2). Therefore, to comprehensively study the role of (p)ppGpp and DksA in Y. enterocolitica, we constructed a DksA-deficient strain (YEND) and knocked out the *relA* and *spoT* genes to obtain a *dksA*-(p)ppGpp double-deficient (YENDRS) strain.

YEND exhibited a longer lag phase and lower growth rate in LB and LBNS media compared to that in the WT while expanding this distinction in the M63 minimum medium; however, it finally reached almost the same biomass. And this impaired phenotype could be complemented by expressing the *dksA* gene in the YEND strain (Fig. 1A, B). Furthermore, it is not surprising that YENDRS cannot grow in the M63 minimum medium because of the absence of (p)ppGpp(Fig. 1C). These results indicated that DksA and (p)ppGpp coregulate the growth of Y. enterocolitica under rich- or low-nutrient conditions.

It has been reported that DksA is required for Salmonella Typhimurium biofilm formation *in vitro*, and its deficiency results in an impaired phenotype, similar to *fliC-fljB* mutant (19). Our study also proved that knocking out the *dksA* gene in Y. enterocolitica weakens biofilm formation ability, and the phenotype can be complemented by expressing pBAD24-*dksA* (Fig. 5). Based on the above results, we concluded that DksA and (p)ppGpp play opposite roles in biofilm formation by Y. enterocolitica. In addition, the double-knockout strain exhibited compromised biofilm-forming ability. Similarly, YEND and YEDRS reduced bacterial EPS synthesis, representing the same tendency as biofilm formation among the strains (Fig. 6).

In the survival assay, our study found that YEND exhibited an even lower cell survival rate than YENRS under redox stress (Fig. 2D). Furthermore, the double-knockout strain further reduced the survival rate of the strains, indicating that (p)ppGpp and DksA coordinately regulate the response to oxidative stress in Y. enterocolitica. Compared with this, YEND exhibited almost the same survival rate as the WT strain after treatment with acid and osmotic stress, suggesting that (p)ppGpp instead of DksA was involved in Y. enterocolitica acid and osmotic stress responses (Fig. 2A, C).

### Disruption of DksA further reduced motility and antibiotic resistance in Y. enterocolitica.

The motility assay and Transmission electron microscopy observation confirmed that DksA positively regulate Y. enterocolitica motility and flagellar synthesis. As shown in Fig. 3, the knockout of DksA could reduce the average swimming diameter from 2.52 cm to 1.20 cm. As expected, the expression of the pBAD24-*dksA* plasmid can complement this phenotype. In addition, YENDRS exhibited the smallest swimming diameter among the strains. Electron micrographs also showed that YENDRS exhibited the least number of 1.7 flagella per cell, which was slightly lower than 2.0 flagella per YEND cell (Fig. 4). These findings indicate that DksA and (p)ppGpp coordinately regulate motility and flagellar synthesis in Y. enterocolitica.

Similar to (p)ppGpp, DksA increased the resistance of *Y. enterocolitis* strains to chloramphenicol and ampicillin. As shown in Table 2, YEND reduced the MIC values for chloramphenicol from 4.0 μg/mL to 0.5 μg/mL, exhibiting a lower dilution than that of (p)ppGpp (1.0 μg/mL). However, the MIC values of YEND were not expanded by further deletion of (p)ppGpp and could be partially complemented by expressing the *dksA* gene. For ampicillin, YEND and YENDRS exhibited the same resistance level of 4 μg/mL, including YENRS. However, neither DksA nor (p)ppGpp affected polymyxin B resistance in *Y. enterocolitis*. These results suggest that both DksA and (p)ppGpp confers chloramphenicol and ampicillin resistance in *Y. enterocolitis*.

### Both (p)ppGpp and DksA are required for motility and biofilm gene expression.

**FIG 7:**
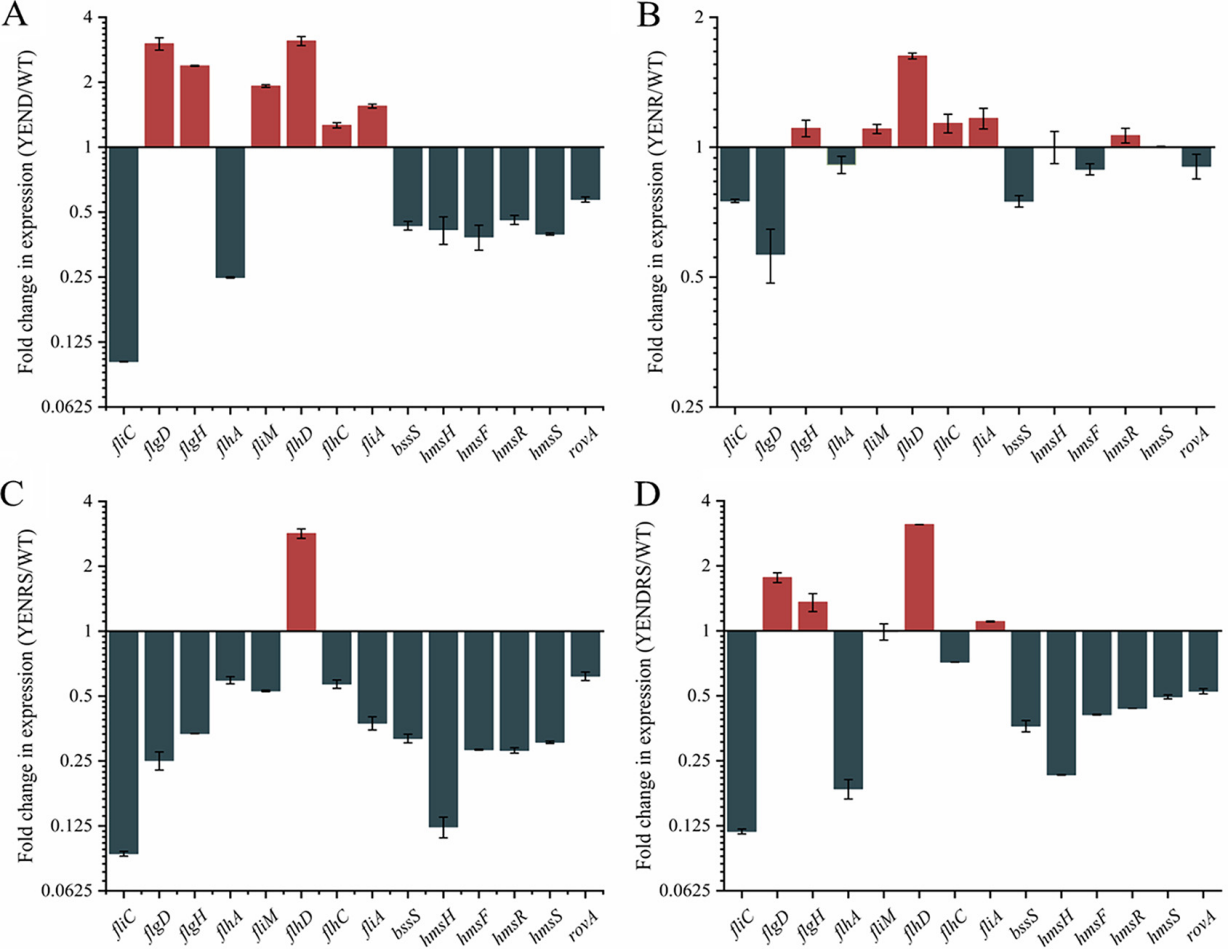
Transcriptional changes in the mutant strains of Y. enterocolitica. Y. enterocolitica were grown to the midlog phase in LBNS medium and the total RNA was extracted. The change in the abundance of the indicated transcripts (normalized to 16sRNA) in the YEND (A), YENR (B), YENRS (C), YENDRS (D) were determined by RT-qPCR. Data are means and SEM from three independent RT-qPCRs.

To better understand how (p)ppGpp and DksA affect motility and biofilm formation, we determined the expression of effector genes during the midlogarithmic growth phase using qRT-PCR (Fig. 7 and Fig. S1B). While it was shown that the transcription of motility-associated genes *flgD*, *flgH*, and *fliM* exhibited an increase in YEND compared to in the WT, the expression of *fliC* and *flhA* was significantly decreased 5- and 10-fold, respectively. Moreover, the transcription of these motility-associated genes was downregulated by 2- to 10-fold in YENRS and exhibited a combined effect in YENDRS. We also analyzed the transcription levels of biofilm-associated genes, including *bssS* encoding biofilm formation regulatory protein and *hmsHFRS* required for the biosynthesis of poly-β-1,6-*N*-acetylglucosamine exopolysaccharide. The results revealed that all these biofilm-associated genes were repressed in the absence of DksA or (p)ppGpp but could be restored when complemented with P_BAD24_- *dksA* or *spoT* plasmids. It is not surprising that the change ratio of motility- and biofilm-associated genes in YENR was more stable than in YENRS since the phenotypes of *Y. enterocolitis* in the absence of RelA also exhibited no significant changes. A previous study reported that RelA positively regulates T6SS through the RovA pathway in Yersinia pseudotuberculosis, which raised our interest in the effect of *Y. enterocolitis* (p)ppGpp and DksA on *rovA* transcription (29). We observed an approximately 2-fold reduction in YEND and YENDRS; however, similar expression levels were observed in the *relA* mutant. These results indicated that (p)ppGpp and DksA are required for motility and biofilm gene expression.

## DISCUSSION

Stringent response is a global regulatory mechanism that is widely present in bacteria and is induced by multiple stresses, such as nutritional starvation and environmental downshift. The signaling molecules (p)ppGpp and DksA act on diverse target genes, resulting in various bacterial phenotypes (36). Their synergistic action has been widely documented, including their direct effects on RNAP activity and indirect effects on RNAP σ-factor activity. However, evidence suggests that (p)ppGpp and DksA may have independent or even opposite effects on phenotypic and gene. One major evidence is the different binding sites of ppGpp and DksA to RNAP. (p) ppGpp can directly bind to RNAP at two opposite surfaces. “Site 1” is located in the amino terminus of the ω subunit and DPBB domain of the β′ subunit, and “Site 2” is at the interface of the secondary channel rim helices of β′, while DksA can only bind to RNAP Site 2 (2, 18, 37). In addition, DksA alone can inhibit transcription to some extent, although (p)ppGpp (when bound to Site 2) greatly enhance the efficiency of DksA inhibition (18). And (p)ppGpp has been shown to directly regulate the transcription of target genes (7). These reasons ultimately lead to (p)ppGpp-deficent and DksA-deficient strains differing in transcriptome and phenotype (5, 6). In this study, comparative studies of the effects of the stringent response regulators DksA and (p)ppGpp on the virulence-related phenotype in *Y. enterocolitic* were performed. It was found that the stringent response of *Y. enterocolitic* strains has a profound effect on pleiotropic phenotypes, including growth, stress survival, motility and biofilm formation, and there are both concerted actions and independent roles in target gene transcription and phenotypes between (p)ppGpp-deficient and DksA-deficient strain.

In this study, it was shown that Y. enterocolitica requires (p)ppGpp to grow in M63 minimal medium rather than in LB rich medium (Fig. 1). Similar results were reported in Erwinia amylovora that (p)ppGpp is necessary for growth in MBMA minimal medium, which is due to the dependence of (p)ppGpp on bacterial *de novo* amino acid synthesis (38). The DksA-deficient strain grew in the M63 minimal medium despite the lower growth rate. Its maximum biomass was not significantly different from that of the WT, indicating that DksA is an auxiliary regulator of stringent response under auxotrophic conditions. Interestingly, there was no remarkable difference between the *relA* deletion mutant and the WT in terms of growth under nutrient starved condition as well as subsequent phenotypic results. This also indicates that the synthetic activity of the remaining SpoT protein from RelA knockout strains is sufficient to provide basal (p)ppGpp under nutrient poor medium conditions, thus maintaining the normal growth of Y. enterocolitica on nutrient starved minimal media. In contrast, the deletion of RelA in S. enterica serovar Typhi result in defective growth under similar under nutrient poor medium (24). These results suggest the differences in stringent response patterns and RSH proteins functions between species-species due to the differences in living conditions and environmental pressure faced. Similar evidence is also provided in *Moraxellaceae* family of gammaproteobacterial, in which that the synthetic activity of SpoT is gradually being lost, indicating that there is an ongoing partitioning of functions for SpoT (8).

In Y. enterocolitica, (p)ppGpp regulates tolerance to acid and hyperosmolar environments, whereas DksA is involved in regulating tolerance to oxidants. A similar study has been previously described in Y. pseudotuberculosis ΔRelA strains. Yang et al. considered that RelA regulates the type VI secretion system (T6SS4) expression through the RovM/RovA pathway, thus generating a stress response to adverse stresses (29). This notion was confirmed in our study, with decreased expression of *rovA* in the (p)ppGpp deficient strain (Fig. 7). Nonetheless, the survival rate of the DksA-deficient strains, which also showed decreased expression of the *rovA* gene, was nearly no different from that of the WT in acidic and high osmotic environments, indicating the existence of other regulators in response to environmental stimuli. Recently, the DksA-DnaJ complex in Salmonella was shown to cooperate with (p)ppGpp to activate RNA polymerase when exposed to hydrogen peroxide, and its regulatory activity differentially modulated by varying hydrogen peroxide concentration (20). In addition, RpoS, a well-known target of (p)ppGpp, has also been shown to be involved in resistance to environmental stress (39). These findings support the perspective that (p)ppGpp contributes to bacterial resistance to adverse stresses in various ways not limited to T6SS4.

Resistance to antibiotics of foodborne pathogens is a serious concern in the food preservation and treatment of infectious diseases. Due to the extensive regulation of metabolism by the stringent response, and the fact that some existing antibiotics work by inhibiting cellular metabolism or inhibiting the synthesis of specific components, (p)ppGpp and DksA are potential targets for developing antibiotic adjuvants. In this study, we found that (p)ppGpp and DksA could confer bacterial resistance to chloramphenicol and ampicillin. A recent study reported that (p)ppGpp is involved in maintaining cysteine homeostasis while inhibiting protein synthesis with chloramphenicol. In general, the intracellular cysteine concentration increased when bacteria were exposed to chloramphenicol, which caused damage to DNA and cellular components owing to the high ability of cysteine to reduce free iron (40). However, on the one hand, (p)ppGpp would inhibit the exportation of glutathione generated by cysteine from cells, thereby reducing the intracellular cysteine level. On the other hand, excess intracellular glutathione can react with hydrogen sulfide, which is also overproduced when exposed to chloramphenicol, thus reducing the damage caused by reducing sulfur. In addition, several studies have explored the relationship between the stringent response and ampicillin resistance, and it is generally accepted that sufficient intracellular levels of (p)ppGpp can shut down cell wall synthesis, thereby inhibiting the action of ampicillin from preventing cell wall synthesis and conferring high bacterial tolerance to ampicillin (26, 41).

Motility and biofilm formation are important for bacterial spread and colonization, thereby affecting pathogenicity and infectivity. In this study, we found that both (p)ppGpp and DksA were able to inhibit flagella formation and attenuate the motility of Y. enterocolitica. However, the regulatory roles of (p)ppGpp and DksA on bacterial motility are controversial, and interpretation of this mechanism requires consideration of both direct and indirect effects of the two signal molecules, as well as the specificity of regulatory mechanisms across species. The effects of (p)ppGpp on flagellar synthesis are almost uniform across in different species. In S. enterica and *X. citri*, deletion of relA and spoT leads to a decrease in bacterial flagellar synthesis and pathogenicity, and E. coli requires (p)ppGpp for flagellar gene activation and chemotaxis (5, 6, 24). Our results also confirmed that the lack of (p)ppGpp inhibited flagella synthesis-related genes such as *fliC*, *flgD*, *flgH*, *flhA*, and *fliM*, resulted in repressed flagella synthesis and weak mobility in *Y. enterocolitis*. However, the effects of DksA on motility is conflicted in recent studies. S. enterica serovar Typhimurium lacking DksA showed defective mobility, possibly due to the inhibition of *fliA*, *fliC*, *cheB*, and *flhD*. Similar results were obtained for Pseudomonas putida and Vibrio cholerae (19, 42, 43). In contrast, the knockout of *dksA* conferred higher expression of chemotaxis and flagella genes than WT strains, resulting in flagella overexpression, hyperflagellated cells, and increased motility in E. coli (23). Justin et al. suggested that this contradictory result may be caused by the soft agar assay method. That is, DksA-deficient strains grow more slowly, which affects the results of assays for strain motility (44). In our study, strains were cultured and assayed in LBNS medium, which hardly affected the growth of mutant strains (Fig. 1). Therefore, the interference of the detection method can be eliminated. Justin et al. also found that DksA directly inhibited the promoters of *flhDC* and *fliA in vitro*, and this was confirmed in our study. Nonetheless, our findings were in contrast to theirs, showing that knockdown of DksA inhibits strain motility (44). We attribute this inhibition of flagella to the decreased expression levels of *fliC* and *flhA* caused by DksA knockout, which may be indirect *in vivo*. Furthermore, biofilm formation was investigated in DksA- and (p)ppGpp-deficient strains, and it was shown that there was an opposite regulatory role in Y. enterocolitica, in which (p)ppGpp inhibited biofilm formation, whereas DksA positively regulated biofilm formation. However, the expression of biofilm genes, such as *bssS* and *hmsHFRS* in (p)ppGpp-deficient strains was suppressed, indicating that that other regulators or mechanisms may be involved in biofilm formation. Liu et al.(27) may explain a similar phenomenon in P. putida KT2440, where they concluded that the deletion of (p)ppGpp reduced the expression level of σ^S^ by affecting transcriptional elongation, resulting in the enhancement of the binding capacity of RNAP and alternative σ factor RpoD. Therefore, the expression of *fleQ*, which is positively regulated by RpoD, increased, and FleQ further enhanced the expression of *lapA* and *bcs*, resulting in enhanced biofilm formation.

**FIG 8:**
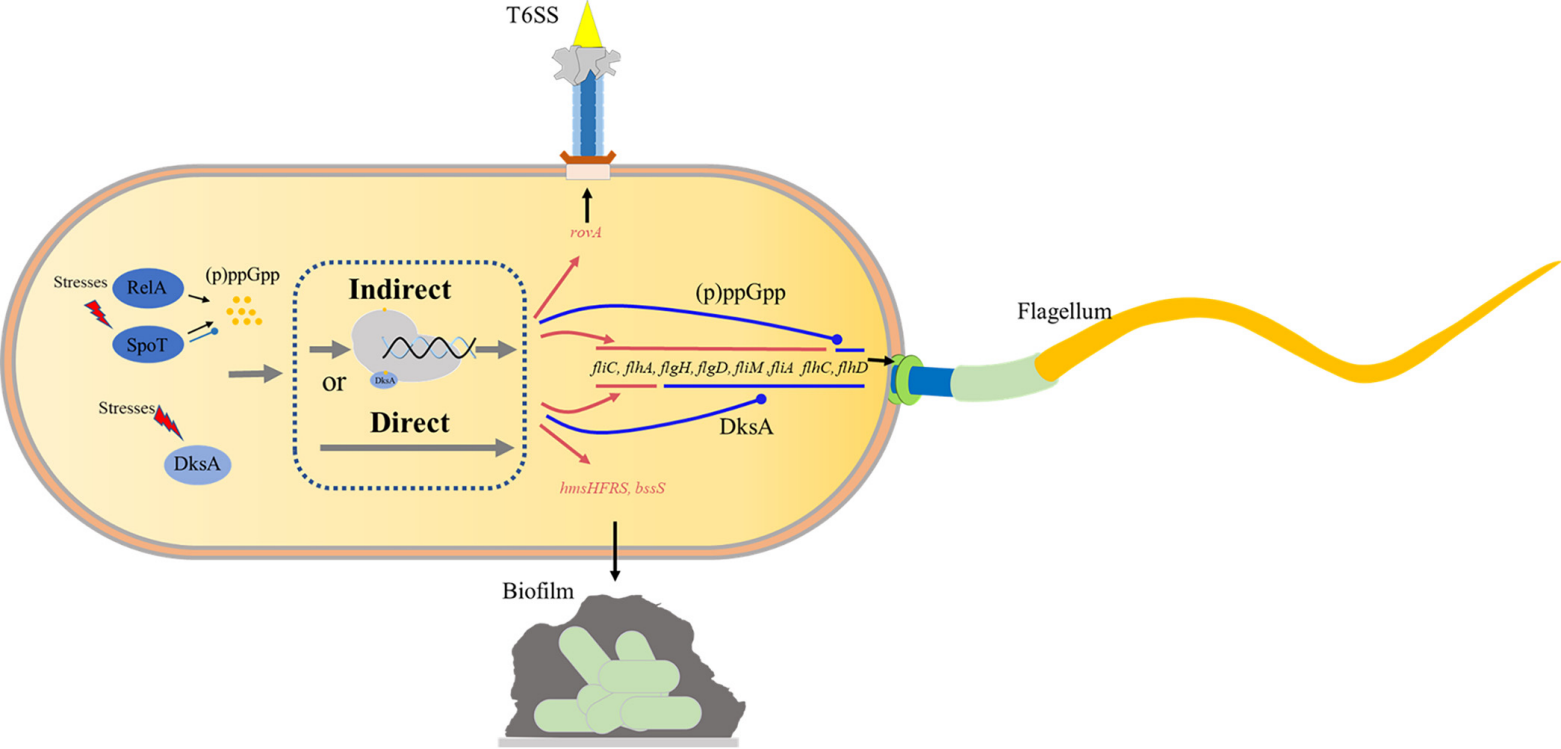
Schematic representations underscoring the possible mechanism(s) of stringent response regulator of Y. enterocolitica *on* pleiotropic phenotype. Red arrows and blue balls indicate positive or negative traits, respectively.

Based on the results of this study and previous observations, a working model of how (p)ppGpp and DksA contribute to Y. enterocolitica regulation of different traits was developed (Fig. 8). (p)ppGpp and DksA exerted a positive effect on the response of Y. enterocolitica to adverse environments, such as antibiotics, low nutrition, low pH, and high osmotic pressure; Furthermore, there are synergistic action on oxidative stress response and cell motility, and opposite action on biofilm formation between (p)ppGpp and DksA, which demonstrated that DksA does not always act as a cofactor to enhance the function of (p)ppGpp in the stringent response, but also exerts transcriptional regulation independently of (p)ppGpp. Together, our investigations revealed the biological function of (p)ppGpp and DksA and their relationship with the response to environmental stress, which is important for understanding the complex mechanism underlying Y. enterocolitica transmissibility and pathogenicity.

## MATERIALS AND METHODS

### Bacterial strains and culture conditions.

The bacterial strains used in the present study are listed in Table 1. E. coli DH5α, used for plasmid construction and amplification, was cultured at 37°C in Luria–Bertani (LB) broth consisting of 5 g/L yeast extract, 10 g/L tryptone, and 5 g/L NaCl. Y. enterocolitica ATCC 23715 (biotype 1B and serotype O:8) was used as the parent strain for the construction of Y. enterocolitica mutants and maintained in LB, defined M63 minimal medium (100 mM KH_2_PO_4_, 15 mM [NH4]_2_SO_4_, 1.8 μM FeSO_4_, 1.0 mM MgSO_4_, and pH 7.0) supplemented with 0.2% (wt/vol) glucose or LBNS (LB without NaCl) at 26°C. In addition, 100 μg/mL ampicillin, 16 μg/mL chloramphenicol, 15 μg/mL cefsulodin, 4 μg/mL irgasan, and 2.5 μg/mL novobiocin were added to the growth medium when appropriate.

### Plasmid construction.

All plasmids used in this study are listed in Table 1, and the primers are listed in Table S1. Upstream and downstream flanking fragments were amplified from the Y. enterocolitica genome for each target gene (*dksA*, *relA*, and *spoT*). A second fusion PCR was performed to connect the two fragments using the forward primer for the upstream fragment and the reverse primer for the downstream fragment. The resultant fragment was digested with XbaI and SacI and cloned into pDS132 to yield suicide plasmids for gene deletion.

To construct plasmids for the complemented strains, a DNA fragment covering the entire coding region was amplified from the Y. enterocolitica genome, digested with two appropriate enzymes, and inserted into pBAD24. The coding gene was expressed by the inducible *araBAD* promoter with the addition of *l-*arabinose in the growth medium.

### Strain construction.

The strategy used to generate the deletion mutants was based on a two-step sucrose counter selection procedure, as described previously (45). Briefly, the suicide plasmid was introduced into E. coli S17-1λpir via electroporation and mobilized into Y. enterocolitica via conjugation. A two-step homologous recombination process was executed, deletion mutant was obtained after PCR verification and gene sequencing. Note that the double mutant YENRS was generated from YENR and the triple mutant YENDRS was generated in the background of YENRS.

### Growth condition.

Fresh overnight bacterial culture was diluted into 250-mL flasks containing 100 mL LB, LBNS, or M63 medium to an OD_600_ of approximately 0.05 and incubated at 26°C and 180 rpm for 30 h. Samples (200 μL) of each strain were taken every 3 h and measured at 600 nm using a microplate spectrophotometer (Puxi University, Co., Ltd., Beijing, China). The experiment was performed in triplicate at least twice, and the error bars represent the standard deviation.

### Survival assay.

Midexponential-phase strains grown in LBNS medium were collected and diluted 50-fold in 20 mL LBNS medium containing 0.5 M NaCl, or HCl (pH 4.0), or NaOH ((pH 10.0) and incubated at 26°C for 1 h, or 1.0 mM H_2_O_2_ for 30 min). Simultaneously, each strain incubated in an LBNS medium without stress was used as the control. The cultures were then diluted 5,000-fold and plated onto LBNS agar plates. The plate colonies were counted after incubating at 26°C for 48 h, and the survival rate was calculated by dividing the number of CFU of stressed cells by the CFU number of the control (29, 46). The experiment was performed at least three times in triplicate, and the error bars represent the standard deviation.

### Motility assay.

Bacterial motility experiments were performed on semisolid LBNS plates containing 0.35% agar. A single colony was inoculated into the LB medium, incubated overnight at 26°C and 180 rpm, and then diluted to OD_600_ = 1.0. In addition, 1 μL of diluted culture was inoculated into the center of swim agar plates supplemented with 0.02 g/L l-arabinose and incubated at 26°C for 48 h without being inverted. The experiment was performed in triplicate at least twice, and the error bars represent the standard deviation.

### Biofilm assay.

Crystal violet was used to quantify the amount of biofilm as previously described (47). Briefly, the overnight bacterial culture was diluted at 1:100 in a 24-well plate containing 1,000 μL LBNS supplemented with 0.02 g/L l-arabinose. LBNS without inoculation was used as blank. After incubation at 26°C for 24 h, 48 h, or 72 h, planktonic cells were discarded by washing with phosphate-buffered saline (PBS), and the formed biofilm was fixed for 2 h at 60°C. Fixed bacteria were stained with 0.1% crystal violet for 10 min. Each tube was washed with water and treated with 1,800 μL 33% acetic acid to release the dye bound to the biofilm, which was then measured at 595 nm using a microplate reader (Puxi University, Co., Ltd.). The experiment was repeated thrice in six replicates, and the error bars represent the standard deviation.

### MIC assay.

The MIC of three antibiotics, polymyxin B, chloramphenicol, and ampicillin, for the eight Y. enterocolitica strains, were determined using a 2-fold broth dilution method (48). Briefly, overnight bacterial cultures grown in LBNS media were diluted to an OD_600_ of 0.1 and then diluted 1,000-fold in a 96-well plate containing 200 μL LBNS supplemented with antibiotics at different concentrations (0, 0.25, 0.5, 1, 2, 4, 8, 16, 32, 64, 128, and 256 μg/mL). LB broth without inoculum was used as the negative control. After incubation at 26°C for 24 h, the lowest concentration at which no visible Y. enterocolitica was detected was defined as the MIC.

### Congo red binding assay.

The Congo red binding assay was performed to evaluate the production of polysaccharides as described previously, with a few modifications (25, 27). Briefly, overnight bacterial cultures grown in LBNS media were diluted to an OD_600_ of 0.01 in a 24-well plate containing 1200 μL LBNS medium. After static incubation at 26°C for 48 h, the bacterial mass and polysaccharides produced by bacterial cells were collected by centrifuging at 10,000 × *g* for 5 min, and the supernatant was discarded. The pellet was resuspended in 600 μL of 100 μg/mL Congo red in 0.9% saline and incubated for 1.5 h at 25°C while it was shaken. The polysaccharide-bound Congo red was then sedimented by centrifuging at 10000 × *g* for 5 min, and the optical density of the supernatant was measured at 490 nm. Finally, the percentage of Congo red was calculated by dividing the optical density of supernatant by the optical density of 100 μg/mL Congo red in 0.9% saline.

### Transmission electron microscopy.

The flagellar morphology of Y. enterocolitica strains grown on LBNS medium was observed using a transmission electron microscope (49). Midexponential culture (2 μL) was dropped onto 200-mesh copper grids. Then the grids were negatively stained with 0.5% phosphotungstic acid for 10 s and fixed for 20 min using hot lamps. Flagellar morphology was visualized using a JEM-1230 electron microscope and the average number of flagella in a single cell was then calculated. The experiment was performed at least twice in triplicate, and similar results were obtained.

### RNA Extraction and RT-qPCR analysis.

RNA was extracted from Y. enterocolitica cultures grown to midlogarithmic phase in LBNS medium using the RNAprep pure Cell/Bacteria kit (Tiangen, China). The extracted RNA was then tested for its concentration and quality using Nanodrop 2000c (Thermo Fisher Scientific, USA) and agarose gel electrophoresis. RNA (200 ng) was subjected to cDNA synthesis using PrimeScript RT Master Mix (TaKaRa, Japan) under the following cycling conditions: 15 min at 37°C and 5 s at 85°C. RT-qPCRs were performed using TB Green *Premix Ex Taq* II (TaKaRa), and 16sRNA was used as a reference for normalization. Data analysis was performed as previously described (50). The reactions were performed in triplicate at least three times.
