# Supplementary material for: Similar and Divergent Roles of Stringent Regulator (p)ppGpp and DksA on Pleiotropic Phenotype of Yersinia enterocolitica
Source: Microbiol Spectr. 2022 Nov 21;10(6):e02055-22. doi: 10.1128/spectrum.02055-22 (PMC9769547; doi:10.1128/spectrum.02055-22)
Supplement: Supplemental file 1 — Table S1 and Fig. S1. Download spectrum.02055-22-s0001.pdf, PDF file, 0.3 MB [file spectrum.02055-22-s0001.pdf]

Supplementary Table 1 Primers used in this study

**Primers name**	**DNA Sequences (5'-3')**	**Application**
dksA-U-F	atatcTCTAGAtaagccgccgatctgctgaat	To generate pDS132-Δ*dksA*
dksA-U-R	gagaagcatccacagcaataatatgtatatcc	To generate pDS132-Δ*dksA*
dksA-D-F	tattgctgtggatgcttctcctacattcat	To generate pDS132-Δ*dksA*
dksA-D-R	ctaatGAGCTCgaagtcaagtcggttacctt	To generate pDS132-Δ*dksA*
spoT-U-F	atatcTCTAGAccgttggcgctgcgcttca	To generate pDS132-Δ*spoT*
spoT-U-R	cgagtctgcccgcctatgaatcctcaacgc	To generate pDS132-Δ*spoT*
spoT-D-F	ttcataggcgggcagactcgctgtctaatt	To generate pDS132-Δ*spoT*
spoT-D-R	gtctcGAGCTCggaaatgacccattatgcag	To generate pDS132-Δ*spoT*
relA-U-F	atatcTCTAGAaacagcaaatcccccaattc	To generate pDS132-Δ*relA*
relA-U-R	gggagaagttaattatctatacccaaagcc	To generate pDS132-Δ*relA*
relA-D-F	atagataattaacttctccctactttgcgac	To generate pDS132-Δ*relA*
relA-D-R	gtctcGAGCTCggtggctcaggctatagcat	To generate pDS132-Δ*relA*
dksA-F	gagacGAATTCatgcaagaagggcaaaaacg	To generate pBAD24-*dksA*
dksA-R	gtctcGTCGACtctagattagcctgccatctgctttt	To generate pBAD24-*dksA*
spoT-F	gagacTCTAGAgtcgacttgtacctgtttgaaagcct	To generate pBAD24-*spoT*
spoT-R	gtctcAAGCTTttaattacggttacggctgac	To generate pBAD24-*spoT*
relA-F	gagacTCTAGAgtcgacatggttgcggtacgaagtgc	To generate pBAD24-*relA*
relA-R	gtctcAAGCTTtcaattgccgtggagccgac	To generate pBAD24-*relA*
16s rRNA-F	gcacgtaatggtgggaactc	qRT-PCR
16S rRNA-R	ctccaatccggactacgaca	qRT-PCR
q-*bssS*-F	atgacgcgatgatgatacgt	qRT-PCR
q-*bssS*-R	tgcttacgacggtcaagatc	qRT-PCR
q-*hmsS*-F	agcatggaaatgacgggaga	qRT-PCR
q-*hmsS*-R	ttaacgaccggtgcaacttc	qRT-PCR
q-*hmsR*-F	gatgatgtaccgcctccaga	qRT-PCR
q-*hmsR*-R	gtgaatagtttcccgcgcat	qRT-PCR
q-*hmsF*-F	tggcaatgccgttaatggag	qRT-PCR
q-*hmsF*-R	cctctggtttggtccagtct	qRT-PCR
q-*hmsH-F*	gccggtcatcacctttccac	qRT-PCR
q-*hmsH-R*	cgaaagataccccgctgcgt	qRT-PCR
q-*dksA*-F	ctcgtccttgagcattctcg	qRT-PCR
q-*dksA*-R	gaataagcttgaaatgcgcc	qRT-PCR
q-*relA*-F	agaagaagtcgctggccttc	qRT-PCR
q-*relA*-R	caggcaaatggcggaaatgg	qRT-PCR
q-*spoT*-F	cgagggtgtctctaaactcg	qRT-PCR
q-*spoT*-R	cctgcaccatcgccatgatc	qRT-PCR
q-*fliC*-F	cagcgtattgggtgcgtctc	qRT-PCR
q-*fliC*-R	gttgcagaatctgtgcacgg	qRT-PCR
q-*flgD*-F	tgggttctatctccggtcag	qRT-PCR
q-*flgD*-R	gtcacaacgccatctttgct	qRT-PCR
q-*flgh*-F	atcagccactgttcgaagac	qRT-PCR
q-*flgh*-R	tcgcattggctgaagagctt	qRT-PCR

q-*flhA*-F	ggtaaacagtcgcagcaacc	qRT-PCR
q-*flhA*-R	tgaacatctgaccaggtggc	qRT-PCR
q-*fliM*-F	ctccatgcgctggagataat	qRT-PCR
q-*fliM*-R	cgggcaaaatcatggtatgg	qRT-PCR
q-*flhD*-F	tggccgatgcattatcgcag	qRT-PCR
q-*flhD*-R	cgtcagatgatgaatcgtat	qRT-PCR
q-*flhC*-F	agccgggggcgtttaattaa	qRT-PCR
q-*flhC*-R	cctcaacaccggtgcattgc	qRT-PCR
q-*fliA*-F	cggtacagcgtatacgtggc	qRT-PCR
q-*fliA*-R	gtccgagccgttgttcaacc	qRT-PCR
q-*rovA*-F	gatcggcgtgcaaagaggat	qRT-PCR
q-*rovA*-R	acttactttgtagttgaata	qRT-PCR

Capital nucleotides represent the restriction enzyme sites.

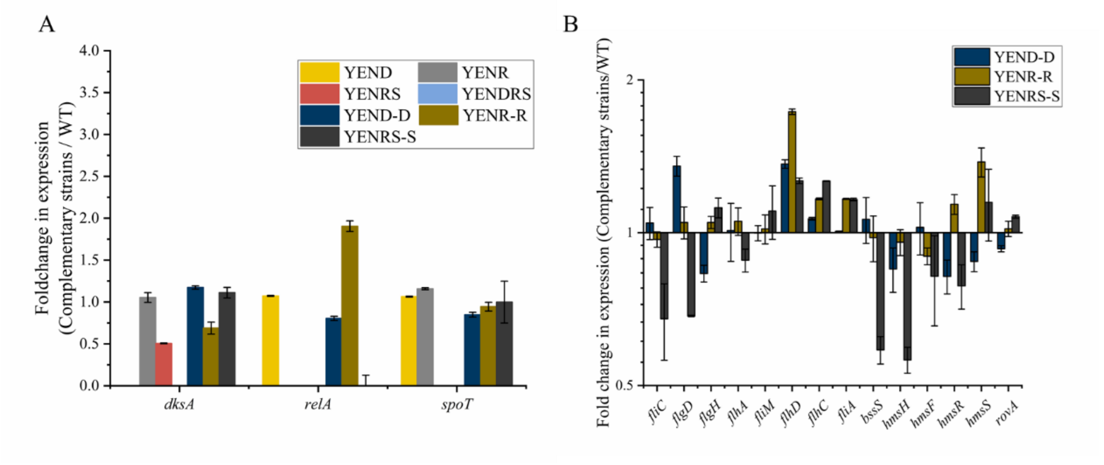

Supplementary Figure 1. Transcriptional changes in the strain of *Y. enterocolitica*. The expression of *dksA*, *relA* and *spoT* in the WT, mutant strains and the complemented strain were showed (A).The changes in the abundance of the indicated transcripts (normalized to 16S RNA) in the complemented strains were determined by RT-qPCR (B). Data are means and SEM from 3 independent RT-qPCRs.
